# Longest terrestrial migrations and movements around the world

**DOI:** 10.1038/s41598-019-51884-5

**Published:** 2019-10-25

**Authors:** Kyle Joly, Eliezer Gurarie, Mathew S. Sorum, Petra Kaczensky, Matthew D. Cameron, Andrew F. Jakes, Bridget L. Borg, Dejid Nandintsetseg, J. Grant C. Hopcraft, Bayarbaatar Buuveibaatar, Paul F. Jones, Thomas Mueller, Chris Walzer, Kirk A. Olson, John C. Payne, Adiya Yadamsuren, Mark Hebblewhite

**Affiliations:** 1 National Park Service, Gates of the Arctic National Park & Preserve, Arctic Inventory and Monitoring Network, 4175 Geist Road, Fairbanks, Alaska 99709 USA; 2 Department of Biology, University of Maryland, College Park, Maryland 20742 USA; 3 National Park Service, Yukon-Charley Rivers National Preserve, Central Alaska Inventory and Monitoring Network, 4175 Geist Road, Fairbanks, Alaska 99709 USA; 4 Norwegian Institute for Nature Research, P.O. Box 5685, Sluppen, NO-7485 Trondheim Norway; 5 Research Institute of Wildlife Ecology, University of Veterinary Medicine Vienna, Vienna, Austria; 6 National Wildlife Federation, Northern Rockies, Prairies, and Pacific Region, 240 North Higgins Avenue, Suite 2, Missoula, Montana 59802 USA; 7 National Park Service, Denali National Park and Preserve, Central Alaska Inventory and Monitoring Network, P. O. Box 9, Denali Park, Alaska, 99755 USA; 8 Senckenberg Biodiversity and Climate Research Centre, Senckenberg Gesellschaft für Naturforschung, Senckenberganlage 25, Frankfurt (Main), Germany; 9 Department of Biological Sciences, Goethe University Frankfurt, Max-von-Laue-Straße 9, 60438 Frankfurt (Main), Germany; 10 Institute of Biodiversity, Animal Health and Comparative Medicine, University of Glasgow, Glasgow, G128QQ United Kingdom; 11 Wildlife Conservation Society, Mongolia Program, Ulaanbaatar, Mongolia; 12 Alberta Conservation Association, 817 4th Avenue South #400, Lethbridge, Alberta T1J 0P3 Canada; 13 Wildlife Conservation Society, Wildlife Health Program, New York, USA; 14 Institute of Remote Sensing and Digital Earth, Chinese Academy of Sciences, No. 9, Dengzhuang South Road, Haidian District, Beijing, 100094 China; 15 Wild Camel Protection Foundation Mongolia, Jukov Avenue, Bayanzurh District, Ulaanbaatar, 13343 Mongolia; 16 Wildlife Biology Program, W. A. Franke College of Forestry and Conservation, University of Montana, Missoula, Montana 59812 USA

**Keywords:** Animal migration, Animal behaviour

## Abstract

Long-distance terrestrial migrations are imperiled globally. We determined both round-trip migration distances (straight-line measurements between migratory end points) and total annual movement (sum of the distances between successive relocations over a year) for a suite of large mammals that had potential for long-distance movements to test which species displayed the longest of both. We found that caribou likely do exhibit the longest terrestrial migrations on the planet, but, over the course of a year, gray wolves move the most. Our results were consistent with the trophic-level based hypothesis that predators would move more than their prey. Herbivores in low productivity environments moved more than herbivores in more productive habitats. We also found that larger members of the same guild moved less than smaller members, supporting the ‘gastro-centric’ hypothesis. A better understanding of migration and movements of large mammals should aid in their conservation by helping delineate conservation area boundaries and determine priority corridors for protection to preserve connectivity. The magnitude of the migrations and movements we documented should also provide guidance on the scale of conservation efforts required and assist conservation planning across agency and even national boundaries.

## Introduction

Long-distance animal migrations (repeated seasonal movement from 1 discrete area to another) are a widespread and highly conspicuous phenomenon in the natural world^1^. The evolutionary mechanisms behind migratory behavior are ecological and biogeographic, and reflect the fitness advantage of seasonally tracking habitats that vary in their distribution of resources, environmental conditions, intra- and inter-specific competition, predation, gene flow, and parasites^2–5^. Resources include high quality forage or surface water, with greater seasonality and variability in vegetative productivity providing impetus for herbivore migration and concomitant benefits of reduced exposure to predation^2,6,7^.

Long-distance, terrestrial migrations are globally imperiled, especially for large mammals, and many have already been extinguished or are under threat^6,8,9^. Loss of migration is cited as a threat to declines of large herbivores worldwide^10^ and once migratory patterns are lost, they may never resume or take decades for populations to relearn^11^. Habitat loss, fences, roads, and other types of human infrastructure and disturbance can act as semi-permeable or impermeable barriers to migration^6,8,12^. Major losses of roadless areas continue^13^ and there are only about 42,000 patches of roadless areas > 100 km^2^ globally^14^. Further, climate change could alter species distributions and migration patterns^15^. Migratory herbivores affect forage biomass, patterns of nutrient diffusion across regions, ecosystem functioning, and predators, which in turn influence decomposers and primary producers^16–18^. Impairment of large herbivore migration can also reduce population sizes^19^, with economic and societal impacts on the people and businesses that benefit from their persistence through harvest or tourism^6,20^.

Two of the most well-known terrestrial migrations are undertaken by caribou (*Rangifer tarandus*) traveling between their winter ranges and calving grounds in the Arctic^21^ and blue wildebeest (*Connochaetes taurinus*) moving between wet and dry seasonal ranges in the Serengeti ecosystem^22^. Caribou are often credited with exhibiting the longest terrestrial migrations on the planet without documentation, or by referencing Fancy *et al*.’s^21^ 5055 km figure (e.g.^8,23^). However, this value was not a migration distance *per se*, but rather the total cumulative distance traveled in a year. Moreover, it was based on currently outdated technology with high positional error, making direct comparisons to current (e.g. GPS) technology problematic. This begs the interesting conservation and management questions: how is migration best measured and which migrations are the longest?

Net squared displacement (NSD) could be used to determine migration distance (e.g.^24^), although it was designed to be a tool to parse behavioral states and the location of greatest displacement may not be a migratory end point (i.e., calving ground or winter range). For example, some Porcupine Herd caribou migrate along non-linear routes that wrap around the east edge of the Brooks Range mountains before turning northwest during their north-bound, spring migration^21^. Thus, maximum NSD does not necessarily occur at the calving grounds. Selection of the start (and end) location(s) have also been shown to greatly impact the results of the NSD-based method (e.g.^25–27^).

A different metric, total cumulative annual distance (TCAD) traveled, which we define as the sum of the distances between successive positional locations over the course of a year, could also be used to assess total migration. Many have misinterpreted Fancy *et al*.’s^21^ results in this manner. While TCAD is a simple, repeatable metric, it is an estimate of movement distance: all movements, not just migratory ones. Thus, it is meaningful and computable for any mobile animal - whether migratory or not. In fact, the TCAD of migratory and non-migratory individuals within the same population can be similar (e.g.^28^).

We suggest that the straight-line, round-trip distance (RTD) between migratory end points is perhaps the most repeatable, versatile, and straight-forward means to measure migration distance. Furthermore, RTD can also be directly compared with historic studies that identify, for example, wintering and calving grounds without recourse to collar-based data. Much like TCAD, RTD varies among individuals within years, among years for the same individual, with changes in population size and density, with differences in seasonal range fidelity, and with new barriers to migration over time^21,29,30^. Reported and historical migration distances are, however, typically reported as a static measurement at the population level. The utility of RTD is constrained to species that display clear migratory patterns but is not applicable to nomadic and resident species^31^.

Obviously, the importance of movement is not just limited to migration. Animals move to acquire food, resources and mates, mark and defend territories, evade predation and parasites, disperse to avoid strife or competition, or increase mating potential^32^. All of these behaviors lie largely outside the migration rubric^1^. How much a particular animal moves depends on its size, trophic level, forage/prey availability, own population density and that of its competition, and on the amount of variability in seasonality, among other factors. We hypothesize that TCAD will be greater for cursorial predators than their prey, as only a limited amount of energy is transferred between trophic levels^33^ and thus these predators must range more widely to track and successfully secure their prey.

In areas with greater seasonal variability, as once-abundant resources become scarce in 1 area, animals must move to another area^3^. Similarly, areas with low primary productivity and/or unpredictable resource availability may force animals to roam more or become nomadic^34^. Thus, we posit that TCAD for larger animals will be less than it will be for smaller animals of the same guild (e.g., grazers), as they can use more ubiquitous, lower-quality forage^35–37^ and may be more resilient to environmental stochasticity due to larger body stores. However, like migration, movement in general has also been adversely affected by human activity and development. For example, Tucker *et al*.^38^ documented that, in large animals, movements were reduced by 50–67% in areas of high human activity. Thus, we predict that TCAD will be greater in more remote, less impacted regions. However, in some cases anthropogenic barriers could force wide-ranging species to move further to find suitable crossing opportunities even in remote areas^39,40^.

Our goals for this paper were to: 1) provide methods to consistently measure migration and movements so that direct comparisons can be made, 2) document the length of RTD migrations for a wide array of species that encompass different regions and ecosystems so that we could determine which species exhibit the longest terrestrial migrations, and 3) determine the TCAD traveled in a year for a suite of animals that varied in movement patterns (migratory, territorial, and nomadic), trophic level, and body size which also had potential to be among the most cursorial large mammals in the world. Lastly, we discuss our results in relation to our hypotheses about trophic levels, vegetative productivity, body size, and human population density in order to explore some of the underlying factors that influence migration and movements.

## Material and Methods

### Study area and literature review

**Figure 1:**
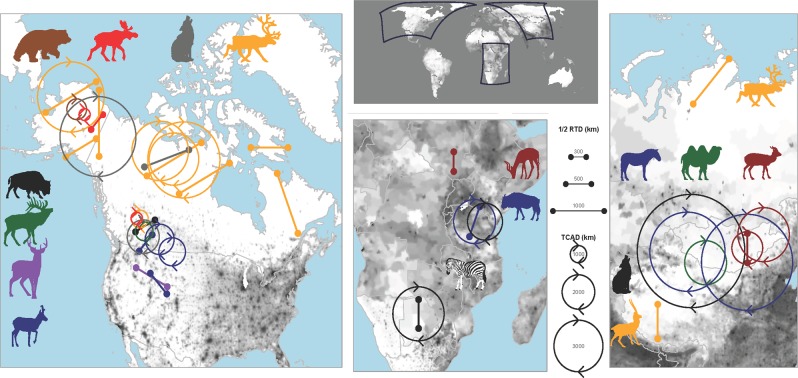
Locations of the study areas of some of the longest, terrestrial migrations and movements in the world. Migration distances are shown as lines of a length equaling half the round-trip distance (RTD), roughly oriented in the direction of the migration for that species. Total annual movement distances (TCAD) are depicted as circles with circumferences equaling how far that species moves in a year. Both RTDs and TCADs are color-coded to match species icons and are scaled to be directly comparable to each other and to all species. They may not match the world backdrop in some locations due to projection issues. Areas with greater human density are shaded darker.

The study area included terrestrial ecosystems worldwide (Fig. 1). We focused our efforts on areas with known long-distance (~300 km) large mammal migrations, a high degree of seasonality, low primary productivity, and/or little human footprint (e.g., the Arctic, central Asia, central and southern Africa, and the inter-mountain and northern great plains regions of North America). We began with existing studies (e.g.^6,8,34^) and then conducted Google Scholar searches using different species name and the term ‘migration’ or ‘movement’. The remaining datasets we collected ourselves.

### Determining migration distances

We used the Euclidean (straight-line), round-trip distance (RTD) between end points to determine length (km) of migrations. The 2 most common sets of migratory end points were winter range and calving area (*e*.*g*., caribou) and wet and dry seasonal ranges (*e*.*g*., wildebeest). We used information found in studies identified during our literature review and with our own datasets. Mostly, the distances were taken exactly as reported in the literature or as double the estimated distances from the far extent of 1 end point (e.g., winter range) to the center of the other end point (e.g., calving area) based on provided range maps. For studies at the population rather than individual level, we measured from the distal portion of the winter (or dry season) range to the center of the calving (or wet season) range.

### Determining total cumulative annual distances

We used GPS datasets that we collected, colleagues made available, or were generally available (e.g., publicly housed in Movebank; www.movebank.org) to estimate total annual distance moved (not as a precise measurement of absolute distance traveled). We defined this distance as the cumulative distance between successive GPS locations over the span of 365 days. Movement rate estimates, like TCAD, are strongly correlated with sample interval^7,41^. Therefore, where possible, we used relocations that were on 8-hour intervals to standardize according to this bias. For datasets collected at shorter intervals (e.g., 1, 2 or 4 hours), we subsampled the data to achieve an 8-hour interval when possible. When we could not obtain an 8-hour interval (e.g., 5- or 12-hour intervals), we determined the total annual distance moved and adjusted it by the negative relationship between movement rates and sampling interval determined for caribou by Joly^41^ (see that study’s Fig. 3). For example, if a wildebeest collar collected data every 12 hours, its calculated total annual movement would be increased by 10%, whereas if it collected data every 5 hours, it would be decreased by 6% (instances noted in the tables). While the relationship between movement rate and sample interval likely varied by species and study area, our method provided an approximate method to standardize movements for direct comparisons. We also assess correlation between TCAD and primary productivity (http://silvis.forest.wisc.edu/data/dhis/)^42^ and human population density (http://sedac.ciesin.columbia.edu/data/collection/gpw-v4/sets/browse) at the center of the range of each species.

### Compliance with guidelines and regulations

All capture, handling, and collaring of study species was in accordance with applicable guidelines and regulations. This includes permitting and institutional animal care and use committee review and approvals from: Alberta Sustainable Resource Development, Fish and Wildlife Research 11861, 16707, 20394, 059-08MHECS-120908, 059-09MHWB-12209; Mongolian Ministry of Nature, Environment and Tourism 5/4275, 5/5656, 6/4136, 6/5621; Montana Fish, Wildlife and Parks 11-2007; National Park Service 2010-1, AKR_GAAR_Gustine_GrizzlyBear_2014, AKR_DENA_Borg_GrayWolf_2016.A3, AKR_YUCH_Sorum_GrayWolf_2016.A3; Saskatchewan Ministry of Environment 09FW040; State of Alaska 07-11, 253217-3, 2012-031R; University of Veterinary Medicine Vienna ETK-15/03/2016; U. S. Geological Survey 2014-01.

## Results

### Migration distances

Caribou and reindeer (also *Rangifer tarandus*) dominated terrestrial migrations in terms of longest RTD (Table 1). They were the only species exceeding 1000 km, with the exception of a population of gray wolves (*Canis lupus*; 1016 km) in the Northwest Territories, Canada that were thought to track caribou and be migratory rather than territorial^43^. There were some outliers of note. First, Alerstam *et al*.^44^ reported a 1200 km, one-way migration for Canadian caribou based on a personal communication. Based on our findings of the same species in the same region and geography of the area, we believe this is probably the round-trip distance. Second, Bekenov *et al*.^45^ reported a 2400 km round-trip migration for 1 population of saiga antelope (*Saiga tatarica*) and 600–1200 km for 2 others.

**Table 1:** Maximum Euclidean (straight-line), round-trip distance (RTD, in km) for different long-distance, terrestrial mammalian migrations.

Species	Location	Distance	Year(s)	References
*Caribou*	Bathurst Herd	1350	1993–2012	^50^
*Caribou*	Porcupine Herd	1350	1985–1987	^21^
*Caribou*	Leaf River Herd	1300	1990–2010	^59^
*Caribou*	Western Arctic Herd	1250	2009–2017	*this study*
*Caribou*	Qamanirjuaq Herd	1250	1993–2012	^50^
*Reindeer*	Taymyr Peninsula	1200	<2003	^60^ in^6^
*Gray wolf*	Northwest Territories	1016	1997–1999	^43^
*Caribou*	South Baffin Island	800	1984–1992	^61^
*Mule deer*	Wyoming/Idaho	772	2017	^62^
*Caribou*	Nelchina Herd	750	1999–2003	^63^
*Tibetan antelope*	Tibet	700	<2005	^64^
*Blue wildebeest*	Serengeti	650	1999–2000	^22,65^
*Mongolian gazelle*	Mongolia	600	2002–2003	^66^
*Burchell’s zebra*	Botswana	588	2007–2013	^67,68^
*Bison*	Alberta	483	<1940	^69^
*Pronghorn*	Alberta/Saskatchewan	435	2003–2010	^27^
*White-eared kob*	South Sudan	400	1980–1982	^3^
*Moose*	Northeast Alaska	392	1995–1996	^70^
*Pronghorn*	Montana/Saskatchewan	315	2003–2010	*this study;*^27^
*Pronghorn*	Wyoming	300	<2005	^71^

### Total annual movement distances

The animal that had the greatest TCAD (7247 km) was a male gray wolf from southwest Mongolia (Table 2). A territorial female wolf with pups from the same area traveled 5429 km in a year. An adult female gray wolf (without pups) from central Alaska moved 5630 km in a year and hunted caribou. An adult male whose pack hunted moose (*Alces alces*) moved 5554 km in a year. Gray wolves from east-central Alaska traveled similar, yet slightly less, distances: a young female gray wolf (without pups) traveled 5116 km in a year, was territorial, and hunted caribou during the winter months. A wolf from this same area dispersed shortly after capture and ended up in the Yukon Territory, Canada. Including the long-distance dispersal, this wolf moved 4686 km in a year. Wolves that primarily hunted moose from this region moved less (maximum = 3131 km/year) than wolves that primarily hunted caribou.

**Table 2:** Maximum total cumulative annual distance (TCAD, in km) traveled by different terrestrial mammals, at or adjusted (denoted with*) to an 8-hr GPS fix rate. Samples sizes (n) for both the number of individuals and animal-years of data, respectively, are also reported.

Species	Location	TCAD	n	Year(s)	References
*Gray wolf*	Southwest Mongolia	7247	2, 3	2003–2005	*this study*;^72^
*Khulan*	Southeast Mongolia	6145	9, 18	2013–2015	*this study*;^51^
Arctic fox*	Northern Canada	5903	12, 24	2008–2009	^56^
*Gray wolf*	Central Alaska	5630	16, 31	2012–2018	*this study*
*Gray wolf*	East-central Alaska	5116	7, 7	2017–2018	*this study*
*Khulan*	Southwest Mongolia	5067	7, 7	2007–2010	*this study*;^52^
*Caribou*	South Slave Beverly/Ahiak	4868	62, 142	2006–2014	*this study*
*Caribou*	Western Arctic Herd	4488	103, 250	2009–2017	*this study*
*Caribou*	Sahtu Bluenose East	3807	47, 91	2005–2014	*this study*
*Mongolian gazelle*	Eastern Mongolia	3464	5, 9	2014–2017	*this study*
*Burchell’s zebra*	Botswana	3456	1, 1	2007–2008	^73,74^
*Caribou*	North Slave Bathurst Herd	3341	28, 38	1996–2014	*this study*
Bactrian camel*	Southwest Mongolia	2821	1, 1	2007–2008	*this study;*^75^
Blue wildebeest*	Serengeti	2819	9, 13	2013–2015	*this study;*^76^
Plains zebra*	Serengeti	2356	6, 6	2007–2008	*this study;*^76^
*Gray wolf*	Alberta	2155	2, 2	2002–2004	*this study*;^77^
Mongolian gazelle*	Southern Mongolia	2080	4, 4	2002–2003	^66^
*Pronghorn*	Alberta/Saskatchewan	1941	38, 38	2006–2007	*this study*
*Pronghorn*	Montana/Saskatchewan	1797	58, 58	2009–2010	*this study*
*Brown bear*	North-central Alaska	1325	30, 56	2014–2016	*this study*
*Elk*	Alberta	1200	4, 4	2003–2004	*this study*;^78^
*Caribou*	Little Smoky Herd	1131	36, 49	1999–2016	*this study*;^79^
*Moose*	Alberta/British Columbia	770	2, 2	2009–2010	*this study*;^80^
*Moose*	North-central Alaska	754	28, 28	2008–2012	*this study*;^28^

Aside from gray wolves, nomadic khulan (*Equus hemionus*; also known as the Mongolian wild ass) exhibited greater TCAD than any other species we analyzed (Table 2). In southeast Mongolia, khulan moved as much as 6145 km/year and as much as 5067 km/year in southwest Mongolia. Caribou in northern North America moved as much as 4868 km/year. No other species besides wolves, khulan, arctic fox (*Vulpes lagopus*) and caribou had total annual movements >3500 km (Table 2). Mongolian gazelle (*Procapra gutturosa*) and Burchell’s zebra (*Equus quagga*) were just below this threshold. Some barren-ground caribou traveled more than 4 times the distance boreal caribou (*e*.*g*., Little Smoky Herd) did in a year (1131 km; Table 2).

**Figure 2:**
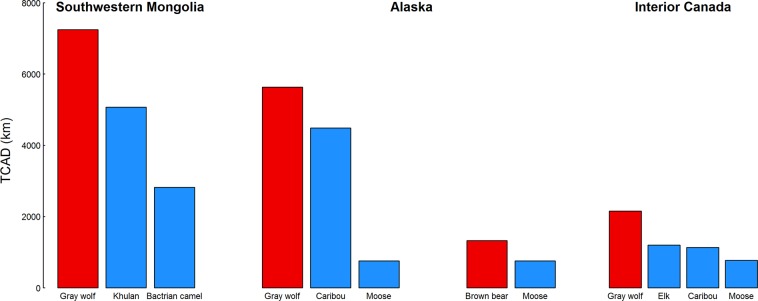
Total cumulative annual distance (TCAD) traveled by predators (red bars) versus their prey (blue bars) from southwestern Mongolia, Alaska and interior Canada.

**Figure 3:**
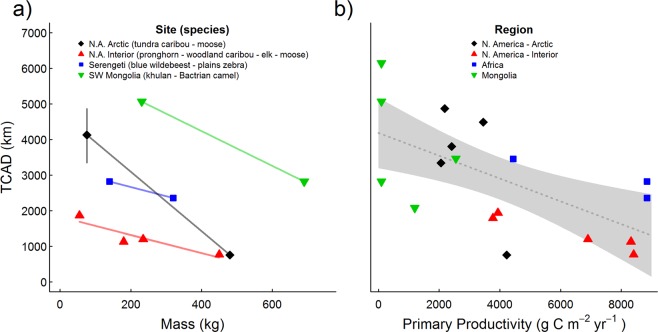
Total cumulative annual distance (TCAD) traveled by herbivores in relation to their (**a**) mass (from Teitelbaum *et al*.^34^ and the authors’ unpublished data; species listed from smallest to largest) and (**b**) primary productivity^42^.

In southwest Mongolia, gray wolves moved more than khulan or wild Bactrian camels (*Camelus bactrianus*; Table 2; Fig. 2), which are among their prey species. In Alaska, gray wolves moved more than their primary prey of caribou or moose. Similarly, in Alberta, gray wolves moved more than elk (*Cervus canadensis*) or boreal caribou. In north-central Alaska, brown bear (*Ursus arctos*) also moved more than moose from the same region. Khulan moved more than camels in southwest Mongolia. This was true for sympatric (unfortunately these data had too many missed GPS fixes for us to report TCAD) and allopatric populations (Table 2, Fig. 3a). In Alberta, pronghorn (*Antilocapra americana*) moved more than elk and caribou, and, in Alaska, caribou moved more than moose. Caribou in northern Canada moved more than those in southern Canada, and pronghorn in Alberta/Saskatchewan moved more than those in Montana/Saskatchewan 400 km to the south. However, moose in northern Alaska had similar annual movement distances as those in southern Canada. TCAD of herbivores was significantly and negatively correlated with primary productivity (Fig. 3b; P < 0.01, F = 10.35, df = 17, R^2^ = 0.393). TCAD was not significantly correlated with human population density.

## Discussion

Caribou have long been thought to exhibit the longest, terrestrial migrations on the planet, albeit often without robust validation. Our study of comparable migratory distances provides substantially more robust support for this claim. With multiple populations with > 1200 km straight-line, round-trip migration distance, caribou are indeed world-class migrators. While we believe caribou likely exhibit the longest extant terrestrial migrations, it is possible that another species may claim that recognition.

In particular, the 2400 km RTD migration reported for saiga antelope in Kazakhstan is by far the longest to our knowledge. However, this distance was estimated without the assistance of tracking technology that allowed known individuals to be relocated^45^ and thus there remains the possibility that Saiga antelope seen on 1 winter range were from different sub-populations than others found on the calving grounds used to determine this distance. Moreover, there have been dramatic declines in saiga antelope populations^46,47^ and populations are currently becoming increasingly constrained by human developments^48^. Thus, saiga antelope migrations have likely been diminished and the species may not exhibit the longest, extant, terrestrial migrations (E.J. Milner-Gulland, *pers*. *comm*.^6^). We encourage the support and publication of GPS-collar research to help identify, quantify and conserve long-distance migrations in this species. Future work quantifying migration distances should focus on movements at the scale of the individual rather than populations for repeatability and comparability.

Migration distance is thought to be positively correlated with population size and density^21,29,30^. We offer 2 more examples that support this hypothesis. First, the Bathurst Caribou Herd, in central Canada, used to migrate up to 1500 km (see^49^), but as the herd has declined, it now migrates about 1350 km^50^. Second, the round-trip migration distance covered by Western Arctic Herd caribou was about 1500 km at its peak population of nearly 500,000 animals (circa 2003), but is now about 1250 km with a population of about half of its peak (*this study*). Recently, however, the Porcupine Herd has shown the opposite trend.

While RTD is a recognizable and valuable metric, it has numerous limitations. Start and end points can be difficult to objectively and quantifiably define and population-level analyses might over-estimated migration distances. Greater standardization and explicit definitions would aid in future comparisons. Additionally, facultative winter migrations, nomadism, dispersal events, territorial and other movements fall outside the domain of RTD. TCAD provides a much more straight-forward, repeatable and quantifiable method to directly compare movements across individuals, populations and species.

Our TCAD results are intriguing, yet preliminary. Predator species consistently moved more than their prey, in line with our hypothesis (Fig. 2). We could not rigorously test our hypotheses due to small samples sizes and biased geography, however, gray wolves from southwest Mongolia moved more than sympatric khulan or wild camels from near the same region. In Alaska, the gray wolf moved more than its primary prey species, caribou or moose, and similarly, in Alberta, the gray wolf moved more than elk or boreal caribou. Also, brown bears moved more than moose in north-central Alaska. While brown bears are omnivores, they prey upon moose and their calves in this region. We suspect this pattern of predators moving more than their prey may be widespread among cursorial predators. We also hypothesize that the TCAD of cursorial predators will increase with more vagile prey or with prey at lower densities and be greater than those for ambush predators. Our findings also suggest that complex social structure, territoriality, and extended periods of relative immobility of young, rather than metabolic and/or anatomical inefficiencies, could prevent wolves and similar cursorial predators from tracking their prey year-round (see^2,5^): not only can they keep up, they can go farther.

In agreement with another of our hypotheses, but also hampered by small sample sizes, smaller members of the same guild appeared to move more than larger members found in the same region (Fig. 3a). For example, in southwest Mongolia, khulan (~230 kg) moved more than wild camels (~690 kg), in Alaska, barren-ground caribou (~90 kg) moved more than moose (~480 kg), in the Serengeti, wildebeest (~140 kg) moved more than zebras (~320 kg), and in Alberta, pronghorn (~55 kg) moved more than elk (~235 kg) and boreal caribou (~180 kg). Again, we suspect this pattern is widespread and related to allometry and the ability of larger species to use lower quality forage than smaller species^35–37^. Our results did, however, document elk moving more than boreal caribou in Alberta. These 2 studies did not overlap spatially and primary productivity was lower in the region where the elk occurred.

We confirmed our prediction that species dwelling in areas with dramatic seasonal variability or low primary productivity (e.g., the Arctic and central Asia) would have greater TCAD (Fig. 3). We expect that low vegetative productivity and high degree of seasonality in southern Mongolia contributed to this region having the greatest TCAD we found^34^. Khulan in southeast Mongolia, where resource availability is more variable, moved more than khulan in southwest Mongolia^51,52^. Zebra in Botswana, where primary productivity is lower, moved more than zebra in the Serengeti. Furthermore, we found wolves in Alaska moved more than wolves found 3000 km south in Alberta, caribou in northern Canada moved more than those in southern Canada, and pronghorn in Alberta/Saskatchewan moved more than those 400 km to the south in Montana/Saskatchewan. We did not find a significant correlation between human population density and TCAD, but that may be related to our focus on species with high TCAD. Indeed, all TCADs > 3500 km were found in areas with densities of < 0.2 people/km^2^. Large patch sizes, associated with low human population density, may also facilitate greater movements.

In our study, gray wolves were the most cursorial species, with individuals from southwest Mongolia exhibiting the longest annual movements. However, like other species (e.g., caribou), wolves displayed a high degree of plasticity. Territorial wolves had greater total annual movements than even long-distance (>450 km) dispersing conspecifics. This may be due to territory maintenance^53^ or hunting behavior. Wolves in packs that hunted caribou moved more than packs that focused on less cursorial moose. Frame *et al*.^54^ reported 1 denning female wolf traveled 341 km in 14 days, to a maximum distance of 103 km from her active den, in pursuit of caribou. Wolves from central Alaska, where caribou density was lower, moved more than wolves from east-central Alaska, where caribou density was greater. While wolves were the most mobile species that we studied, they modulate movements in relation to their primary prey^55^. Meso-carnivores inhabiting low productivity regions, such as the arctic fox, warrant more attention as they can travel very long distances (>4400 km) in 4 or 5 months^56,57^ as well as annually (>4900 km based on daily or every other day PTT relocations^56^, also see Table 2).

While caribou exhibited the longest migration distances, these did not translate into the greatest annual distances traveled. Caribou were outpaced annually by nomadic khulan, but also by their primary predator, the gray wolf and the arctic fox. Caribou, from various study areas, were the next most cursorial species. We posit that extreme movements, in caribou and other species that travel great distances, are driven by extreme seasonal variability, patchy distribution of key resources, low primary productivity, predator avoidance, territoriality of conspecifics, and/or access to parturition location.

## Conclusion

Our work documents and compares some of the longest, extant terrestrial migrations and movements in the world in a standardized way. Such long-distance movements are increasingly challenged by infrastructure development that hinders mobility^38^, thus data on long-distance migrations will help future efforts to identify key areas and species of conservation concern, as well as a better understanding of the mechanisms that structure overall movement of large terrestrial mammals. Furthermore, the spatial and temporal scale of these movements should be accounted for when conservation, corridor, and mitigation planning is undertaken. Territorial, migratory, and nomadic species populations may require different conservation planning strategies^58^. Conservation of long-distance terrestrial movements should also have ancillary benefits to a wide array of other species and ecosystem services.

## Data Availability

Data generated during this study are included in this published article (see Tables 1 and 2). Underlying GPS data used to generate these results may made available from the corresponding author on a case by case basis, in conjunction with the researcher(s) managing those datasets, and in accordance with respective legal constraints.
