# Author Correction: Longest terrestrial migrations and movements around the world

**DOI:** 10.1038/s41598-020-57446-4

**Published:** 2020-01-15

**Authors:** Kyle Joly, Eliezer Gurarie, Mathew S. Sorum, Petra Kaczensky, Matthew D. Cameron, Andrew F. Jakes, Bridget L. Borg, Dejid Nandintsetseg, J. Grant C. Hopcraft, Bayarbaatar Buuveibaatar, Paul F. Jones, Thomas Mueller, Chris Walzer, Kirk A. Olson, John C. Payne, Adiya Yadamsuren, Mark Hebblewhite

**Affiliations:** 1 National Park Service, Gates of the Arctic National Park & Preserve, Arctic Inventory and Monitoring Network, 4175 Geist Road, Fairbanks, Alaska 99709 USA; 2 Department of Biology, University of Maryland, College Park, Maryland, 20742 USA; 3 National Park Service, Yukon-Charley Rivers National Preserve, Central Alaska Inventory and Monitoring Network, 4175 Geist Road, Fairbanks, Alaska 99709 USA; 4 Norwegian Institute for Nature Research, P.O. Box 5685, Sluppen, NO-7485 Trondheim, Norway; 5 Research Institute of Wildlife Ecology, University of Veterinary Medicine Vienna, Vienna, Austria; 6 National Wildlife Federation, Northern Rockies, Prairies, and Pacific Region, 240 North Higgins Avenue, Suite 2, Missoula, Montana 59802 USA; 7 National Park Service, Denali National Park and Preserve, Central Alaska Inventory and Monitoring Network, P. O. Box 9, Denali Park, Alaska, 99755 USA; 8 Senckenberg Biodiversity and Climate Research Centre, Senckenberg Gesellschaft für Naturforschung, Senckenberganlage 25, Frankfurt (Main), Germany; 9 Department of Biological Sciences, Goethe University Frankfurt, Max-von-Laue-Straße 9, 60438 Frankfurt (Main), Germany; 10 Institute of Biodiversity, Animal Health and Comparative Medicine, University of Glasgow, Glasgow, G128QQ United Kingdom; 11 Wildlife Conservation Society, Mongolia Program, Ulaanbaatar, Mongolia; 12 Alberta Conservation Association, 817 4th Avenue South #400, Lethbridge, Alberta T1J 0P3 Canada; 13 Wildlife Conservation Society, Wildlife Health Program, New York, USA; 14 Institute of Remote Sensing and Digital Earth, Chinese Academy of Sciences, No. 9, Dengzhuang South Road, Haidian District, Beijing, 100094 China; 15 Wild Camel Protection Foundation Mongolia, Jukov Avenue, Bayanzurh District, Ulaanbaatar, 13343 Mongolia; 16 Wildlife Biology Program, W. A. Franke College of Forestry and Conservation, University of Montana, Missoula, Montana 59812 USA

Correction to: *Scientific Reports* 10.1038/s41598-019-51884-5, published online 25 October 2019

This Article contains errors in the Results section under subheading ‘Total annual movement distances’.

“In southwest Mongolia, gray wolves moved more than khulan or wild Bactrian camels (*Camelus bactrianus*; Table 2; Fig. 2), which are among their prey species.”

should read:

“In southwest Mongolia, gray wolves moved more than khulan or wild Bactrian camels (*Camelus ferus*; Table 2; Fig. 2), which are among their prey species.”

Additionally, in Table 2, “Bactrian camel” should read “Wild camel”.

